# Tale of the Breslow intermediate, a central player in N-heterocyclic carbene organocatalysis: then and now

**DOI:** 10.1039/d1sc01910d

**Published:** 2021-05-11

**Authors:** Monika Pareek, Yernaidu Reddi, Raghavan B. Sunoj

**Affiliations:** Department of Chemistry, Indian Institute of Technology Bombay Powai Mumbai 400076 India sunoj@chem.iitb.ac.in

## Abstract

N-Heterocyclic carbenes (NHCs) belong to the popular family of organocatalysts used in a wide range of reactions, including that for the synthesis of complex natural products and biologically active compounds. In their organocatalytic manifestation, NHCs are known to impart umpolung reactivity to aldehydes and ketones, which are then exploited in the generation of homoenolate, acyl anion, and enolate equivalents suitable for a plethora of reactions such as annulation, benzoin, Stetter, Claisen rearrangement, cycloaddition, and C–C and C–H bond functionalization reactions and so on. A common thread that runs through these NHC catalyzed reactions is the proposed involvement of an enaminol, also known as the Breslow intermediate, formed by the nucleophilic addition of an NHC to a carbonyl group of a suitable electrophile. In the emerging years of NHC catalysis, enaminol remained elusive and was largely considered a putative intermediate owing to the difficulties encountered in its isolation and characterization. However, in the last decade, synergistic efforts utilizing an array of computational and experimental techniques have helped in gaining important insights into the formation and characterization of Breslow intermediates. Computational studies have suggested that a direct 1,2-proton transfer within the initial zwitterionic intermediate, generated by the action of an NHC on the carbonyl carbon, is energetically prohibitive and hence the participation of other species capable of promoting an assisted proton transfer is more likely. The proton transfer assisted by additives (such as acids, bases, other species, or even a solvent) was found to ease the kinetics of formation of Breslow intermediates. These important details on the formation, *in situ* detection, isolation, and characterization of the Breslow intermediate are scattered over a series of reports spanning well over a decade, and we intend to consolidate them in this review and provide a critical assessment of these developments. Given the central role of the Breslow intermediate in organocatalytic reactions, this treatise is expected to serve as a valuable source of knowledge on the same.

## Abstract 2

Molecular insights on the formation, detection, and even isolation of the Breslow intermediate, which is the most important species in N-heterocyclic carbene (NHC) catalysis, as obtained from experimental and computational studies, are presented.

## Graphical abstract

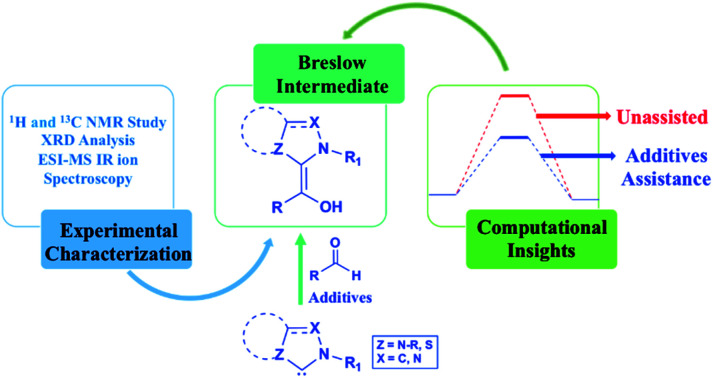

## Introduction

The topic of N-heterocyclic carbenes (NHCs) has been richly reviewed in recent years, with most of those reviews narrating their translational impact from a fleeting intermediate to a versatile organocatalyst for an unprecedented array of reactions.^1^ The key premise in NHC catalysis is the formation and the involvement of an enaminol intermediate, known as the Breslow intermediate. Albeit being scattered, many pieces of experimental evidence and a good number of computational studies on the generation of Breslow intermediates in NHC catalyzed transformations can be found in the literature. The lacuna in the burgeoning area of NHCs is the lack of a compendium on Breslow intermediates, focusing on their formation, detection, and isolation. It is therefore quite timely to give these aspects of NHCs their deserving attention in the form of a review article.

**Scheme 1:**
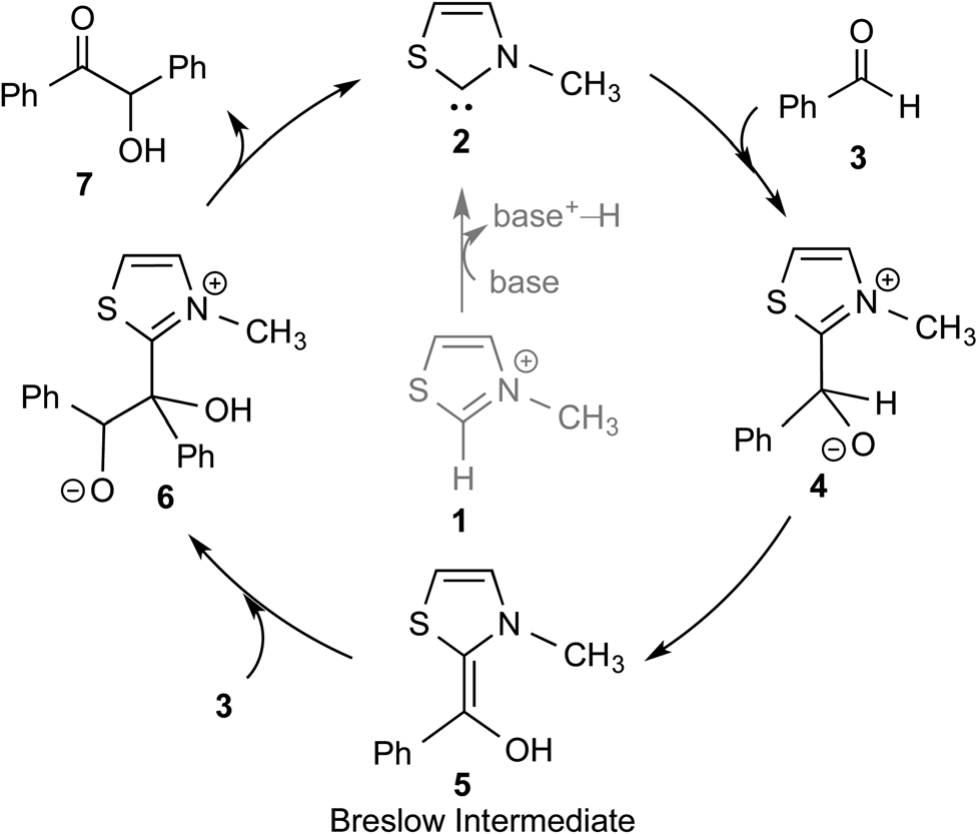
A plausible mechanism of thiazolium catalyzed benzoin reaction as proposed by Breslow.

The textbook concept of umpolung,^2^ which signifies reversal of polarity, is utilized in several organic reactions. The carbonyl carbon of an aldehyde is an electrophilic site, which upon reaction with a cyanide ion can be made nucleophilic, effectively leading to a reversal of polarity. More than a century ago, Wöhler and Liebig discovered the homodimerization of aldehydes in the presence of cyanide ions that leads to benzoin products.^3^ The Lapworth mechanism for such cyanide catalyzed benzoin reaction contributed to the early rationalization of umpolung reactivity.^4^ Decades later, Ukai and co-workers observed that thiazolium salts are also capable of catalyzing the benzoin reaction.^5^ In a landmark article in the year 1958, Breslow proposed a mechanism, as shown in Scheme 1, for thiazolium catalyzed benzoin reaction.^6^ The thiazolium catalyst precursor **1** is deprotonated at the most acidic position by the action of a base to generate carbene **2**. The nucleophilic carbene **2** can then add to the electrophilic benzaldehyde **3** to form a zwitterionic intermediate, **4**. A critical proton transfer from the aldehydic carbon to the geminal oxygen atom can then provide the diamino enol intermediate **5**, which in more recent years came to be known as the Breslow intermediate. The nucleophilic Breslow intermediate can then combine with a second molecule of benzaldehyde to form the benzoin product (**7**) with the expulsion of the free carbene. While the formation of the diamino enol intermediate was suggested quite a few years ago, renewed attention to such intermediates was triggered due to the emergence of NHCs as promising organocatalysts in recent times.^1,9^

It is interesting to note that even decades before the transformative phase of NHCs, from an esoteric species to a ubiquitous catalyst, efforts on their isolation by Wanzlick and others led to dimerized products. The ability of such dimers to dissociate and to react with different electrophiles offered some early signs of promise toward developing the reactivity of NHCs.^7^ In a seminal report back in 1991, Arduengo and co-workers disclosed the crystal structure of an isolable *N*-adamantyl substituted NHC. Conspicuously, a chemist's ability to ‘see’ the molecular structure of a free NHC, which otherwise remained an elusive intermediate back then, served as an impetus to subsequent developments in this domain.^8^ In particular, the ensuing years witnessed an unprecedented growth in the use of NHCs as organocatalysts for a gamut of organic transformations. Especially, in such NHC catalyzed reactions, there has been the implicit assumption of participation of the Breslow intermediate. As one reads this article, it will become increasingly clear how systematic efforts helped researchers in taming this fleeting intermediate and eventually to make it amenable to detection and characterization.

**Scheme 2:**
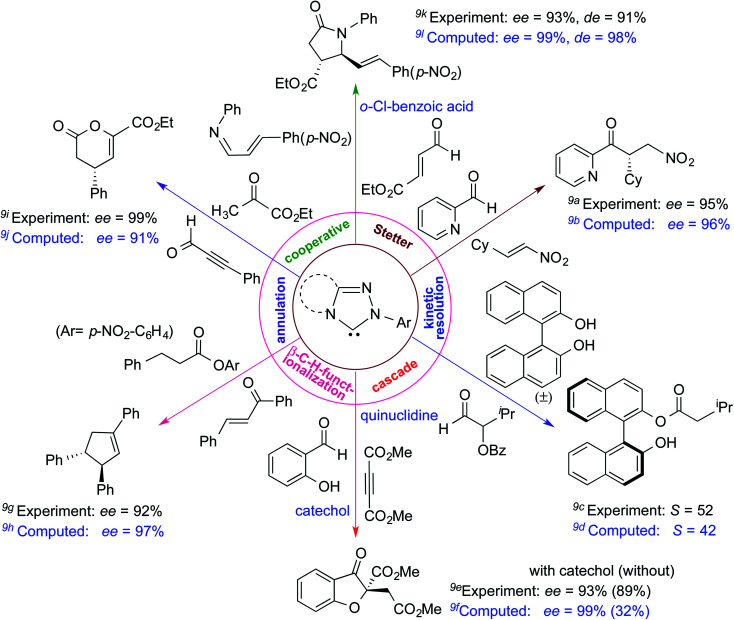
A representative set of chiral NHC catalyzed asymmetric transformations and a succinct comparison of the experimental enantioselectivities with those obtained using density functional theory (M06-2X) computations (ref. 9).

The formation of the Breslow intermediate, in transient or detectable forms, is vital to the catalytic applications of NHCs. Different reaction conditions have been employed in NHC catalysis, particularly due to their higher-level utilization such as that in asymmetric transformations. Computational studies were effectively employed in gaining valuable molecular understanding on the origin of stereoselectivities in such reactions, as evident from a representative set of examples shown in Scheme 2.^9^ These NHC catalyzed asymmetric transformations not only demonstrate their versatile role in diverse range of reactions, but also convey the synergism of density functional theory computations in faithfully reproducing the experimental enantioselectivities. One of the vital details encompassing the whole domain of NHC organocatalysis that demands attention is the mechanism of formation of Breslow intermediates. In the following section, an overview of the current understanding on the formation of Breslow intermediates under different catalytic conditions that vary in terms of the presence/nature of certain types of additive is provided.

## Mechanistic insights on the formation of the Breslow intermediate

**Scheme 3:**
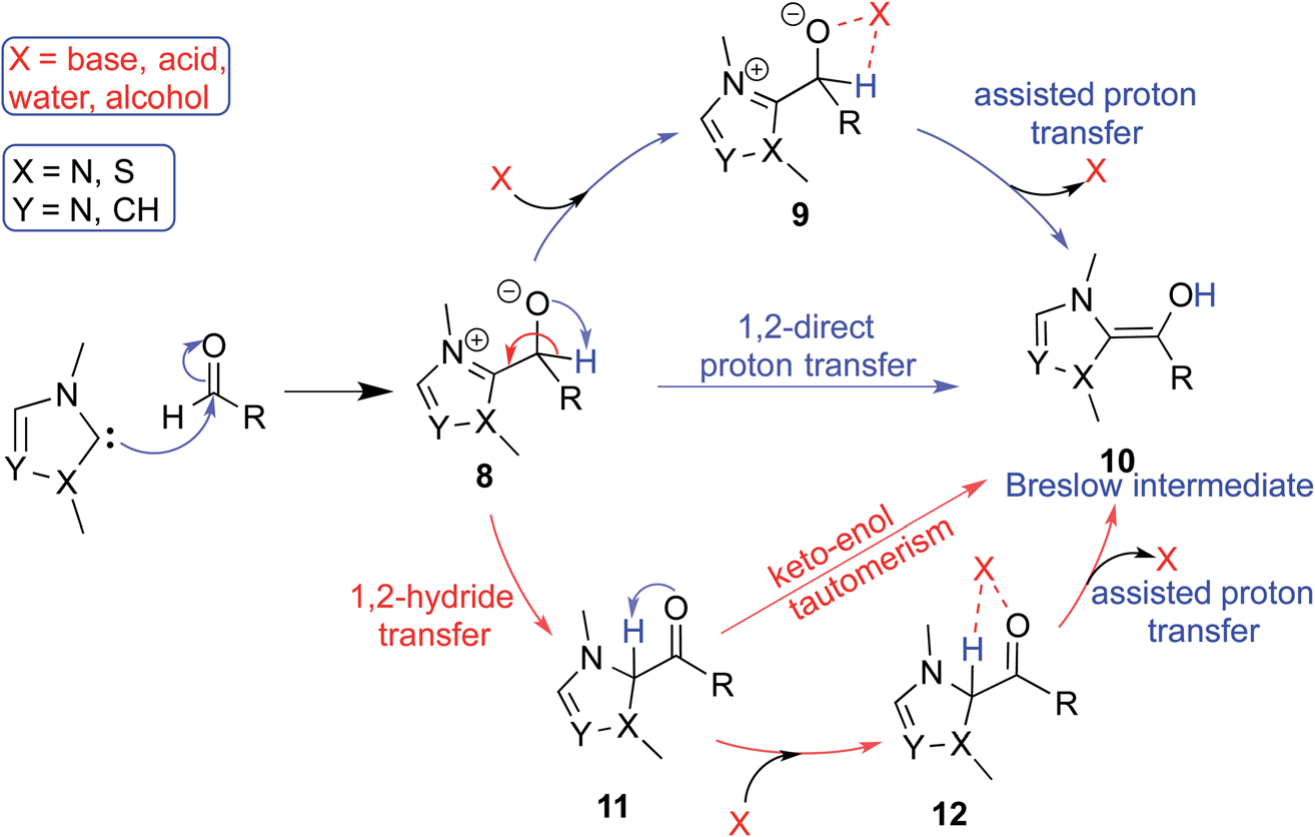
A general mechanism for the formation of the Breslow intermediate under different likely reaction conditions.

In the generally accepted mechanism, nucleophilic addition of the NHC to an electrophilic reaction partner (*e.g.*, aldehyde, enal, *etc.*) generates a zwitterionic intermediate (**8**) first (Scheme 3). A critical proton transfer in this intermediate, through some of the pathways described below, can then lead to the Breslow intermediate. A large body of literature remains surprisingly silent about the feasibility of such 1,2-proton transfer whilst making implicitly general assumptions that a Breslow intermediate is readily formed. The first possibility is a direct proton transfer in the zwitterionic intermediate (**8**) *via* a highly strained three-membered transition state. In the second alternative, the zwitterionic intermediate undergoes a 1,2-hydride transfer to form the keto intermediate (**11**) followed by a tautomerization to the Breslow intermediate (**10**), again *via* a strained four-membered transition state. Along the expected lines, the computed barriers for the 1,2-direct proton transfer in **8** to form **10** are generally found to be very high, indicating that this pathway is not feasible (column 5, Table 1).^10^ To circumvent the strained geometries of the proton transfer transition state, explicit participation of other available molecules from the immediate environment has been proposed.^11^

**Table 1:** A selected list of electrophiles and NHCs known to form Breslow intermediates under different reaction conditions and the corresponding computed elementary step barriers (Δ*G*^‡^/Δ*E*^‡^ in kcal mol^−1^) for the unfavorable 1,2-direct proton transfer

Reaction no.	Electrophile	NHC	Reaction conditions	Δ*G*^‡^
R1	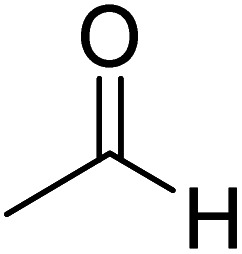	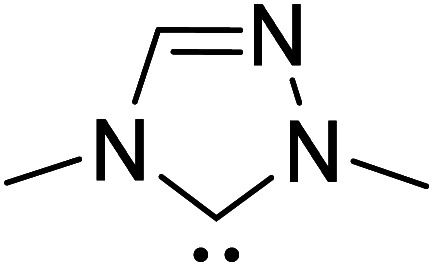	rt	39.1
R2	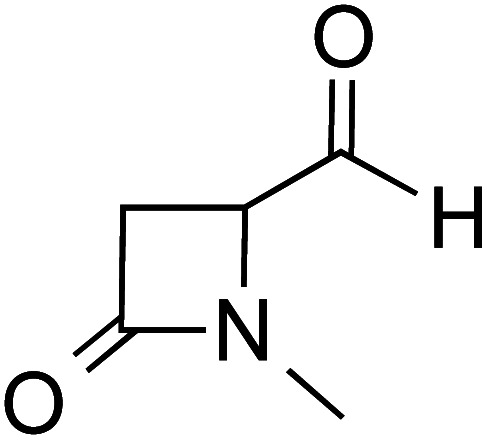	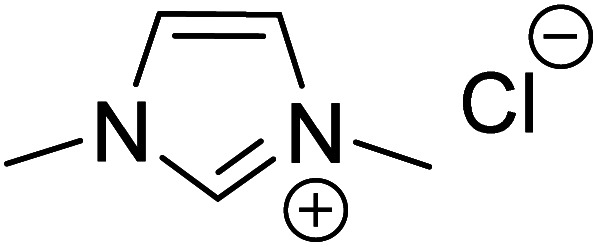	DBU, CH_2_Cl_2_, rt	44.0
R3	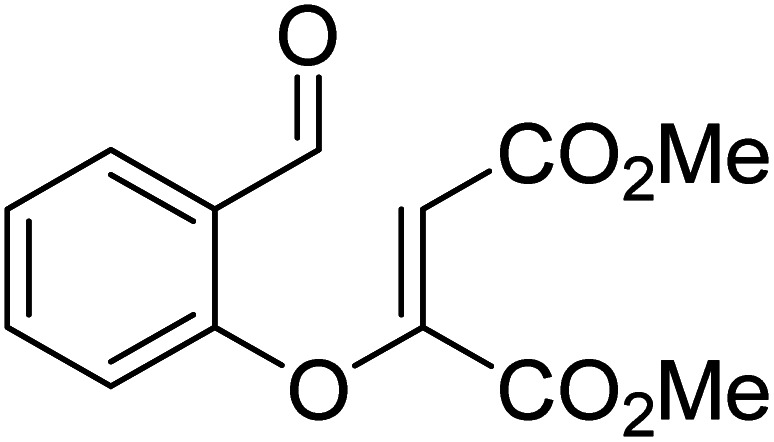	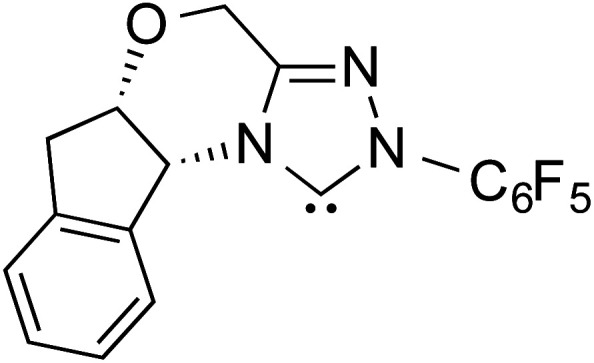	Quinuclidine, toluene, catechol, rt	48.0
R4	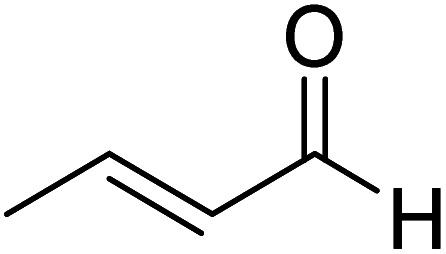	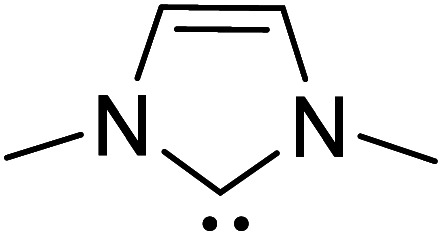	DBU, THF, rt	46.0
R5	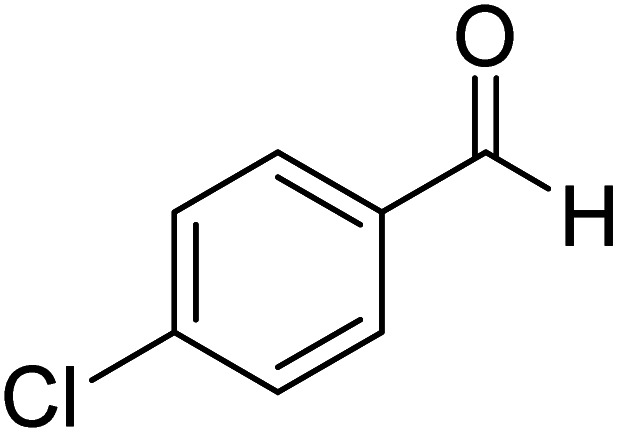	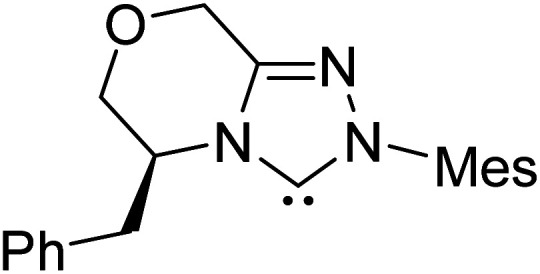	KO^*t*^Bu, toluene, 0 °C	52.8
R6	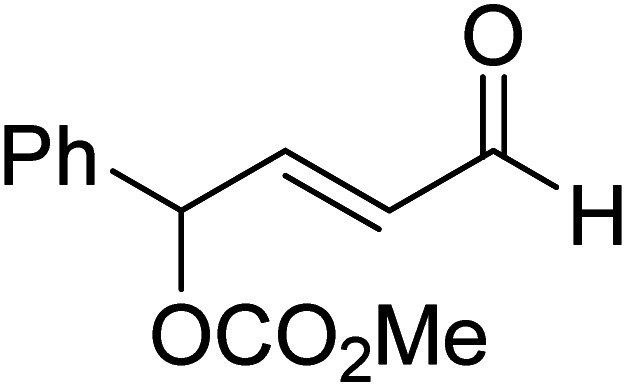	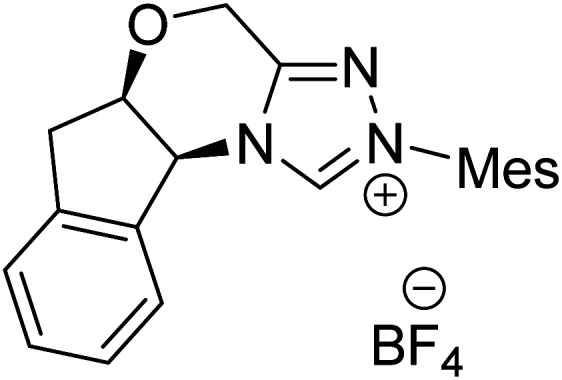	K_2_CO_3_, THF, rt	42.4
R7	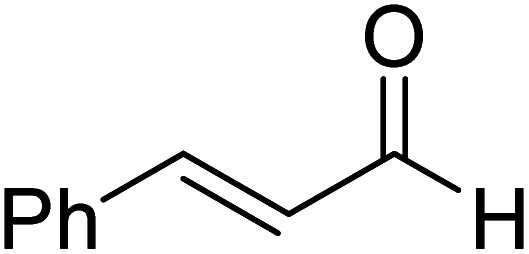	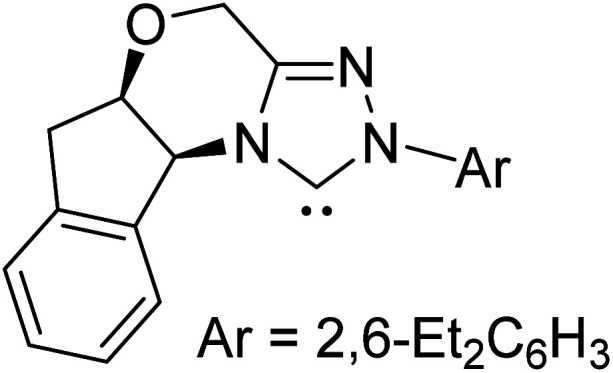	LiCl, DBU, THF, rt	46.4
R8	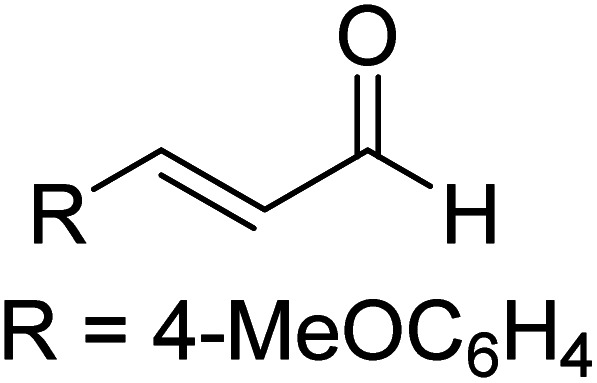	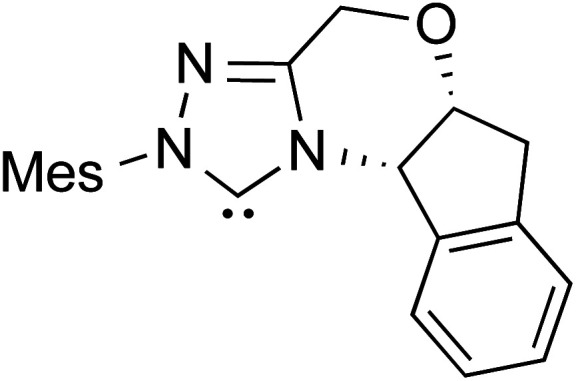	AcOH, THF, rt	36.1 (Δ*E*^‡^)
R9	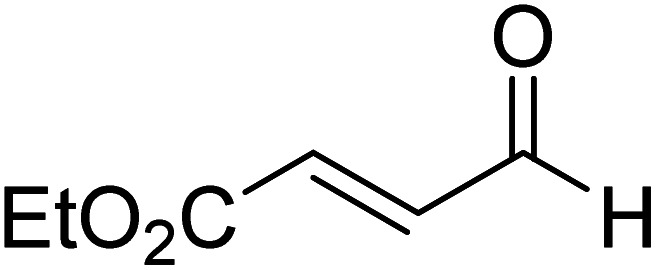	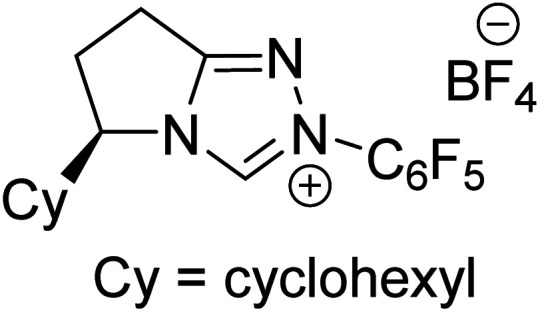	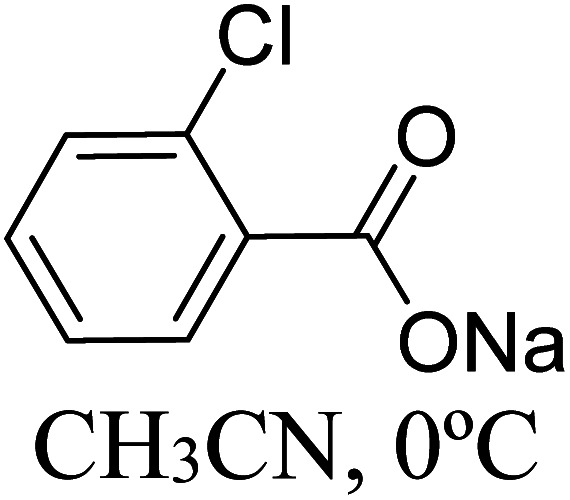	47.1
R10	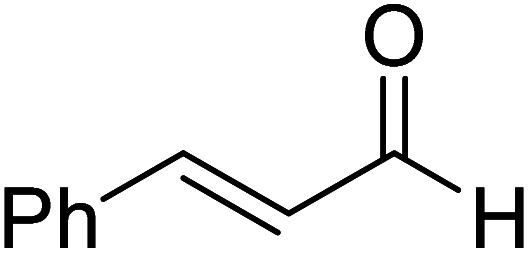	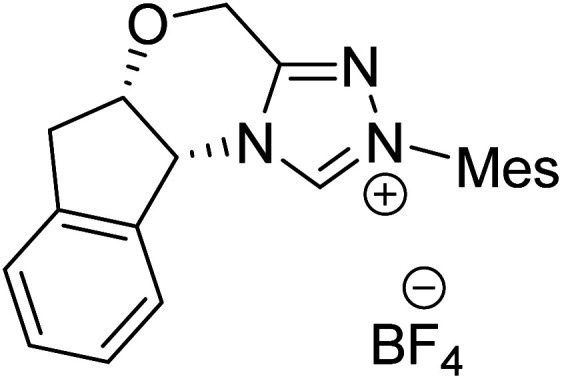	TMEDA, DCM, rt	44.3

An assisted proton transfer invokes a more direct role for (i) the base present in the reaction mixture (*e.g.*, DBU, ^i^Pr_2_NEt, K_2_CO_3_), (ii) Brønsted acids, or (iii) other protic additives (*e.g.*, H_2_O, MeOH, PhOH, catechol). There have been interesting density functional theory studies demonstrating the role of different additives in the formation of Breslow intermediates. In this section, we provide the energetic details on the formation of Breslow intermediates using a few representative examples as listed in Table 1.^10^ Note that most of our discussions in the latter sections make use of the reactions listed in this table.

### (A) Intermolecular proton transfer pathway

**Scheme 4:**
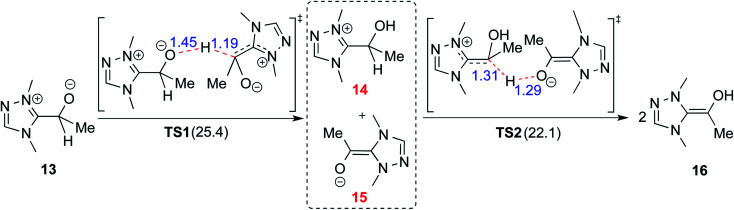
Intermolecular proton transfer between two molecules of zwitterionic intermediate **13** and then between the resulting ion-pair intermediates (**14** and **15**) leading to the formation of the Breslow intermediate (**16**). The activation barriers (Δ*G*^‡^ in kcal mol^−1^) are provided in parentheses.

In 2008, Yates and co-workers employed density functional theory computations to study the mechanism of a Stetter reaction between acetaldehyde and a Michael acceptor (R1, Table 1).^10*a*^ The proposed mechanism for the formation of the Breslow intermediate (**16**) is shown in Scheme 4. The most vital aspect that demands careful attention is the proton transfer in the initially formed zwitterionic adduct (**13**) between the NHC and acetaldehyde. A likely two-step bimolecular proton transfer between intermediates **14** and **15** was invoked.^10*a*,*b*^ An overall activation barrier of 25.4 kcal mol^−1^ was found at the B3LYP/6-311+G(2d,p) level of theory, which is 13.7 kcal mol^−1^ lower than that for the unassisted 1,2-proton transfer in **13** (39.1 kcal mol^−1^).^10*a*^ While this pathway appears energetically reasonable, it is instructive to consider that an encounter between two activated species (such as the NHC–aldehyde adduct) under the reaction conditions is less likely than that between the zwitterionic intermediate and any other molecule abundantly available in the medium. Hence, alternative routes for the generation of the Breslow intermediate need to be considered (*vide infra*).

### (B) Alcohol assisted pathway

**Fig. 1:**
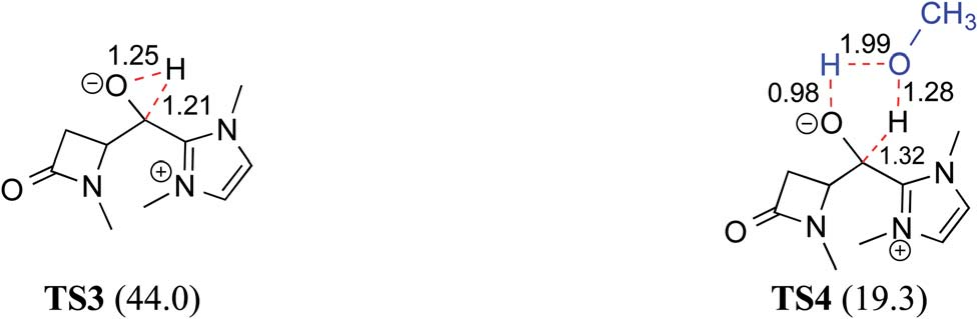
The key geometric features of 1,2-direct proton transfer and methanol assisted proton transfer transition states for the formation of the Breslow intermediate. The activation barriers (Δ*G*^‡^ in kcal mol^−1^) at the PCM_(DCM)_/B3LYP/6-31G** level of theory are given in parentheses. Distances are in angstroms.

The presence of protic additives is known to be beneficial in certain reactions as they can facilitate easier proton transfer.^10*c*,12^ Interesting examples were reported wherein methanol, catechol, *etc.* assisted the critical proton transfer leading to the formation of the Breslow intermediate.^9*f*,10*c*^ As part of their mechanistic investigation on NHC catalyzed ring expansion of 4-formyl-β-lactam (R2, Table 1), Domingo and co-workers reported a barrier of 44.0 kcal mol^−1^ for 1,2-direct proton transfer *via***TS3** at the PCM_(DCM)_/B3LYP/6-31G** level of theory (Fig. 1).^10*c*^ On the other hand, a methanol assisted proton transfer *via***TS4** was noted to exhibit a much lower barrier. In the proton transfer transition state, the explicitly included methanol donates its proton to the alkoxy oxygen of the zwitterionic intermediate and concomitantly abstracts the aldehydic C–H proton. The activation barrier for this concerted methanol-assisted proton transfer (**TS4**) was 24.7 kcal mol^−1^ lower than that for the unassisted pathway *via***TS3**.^10*c*^ Diminished strain in the transition state geometry as well as the additional specific interaction with the methanol can be considered as the origin of the lower barrier in the methanol assisted pathway.

**Scheme 5:**
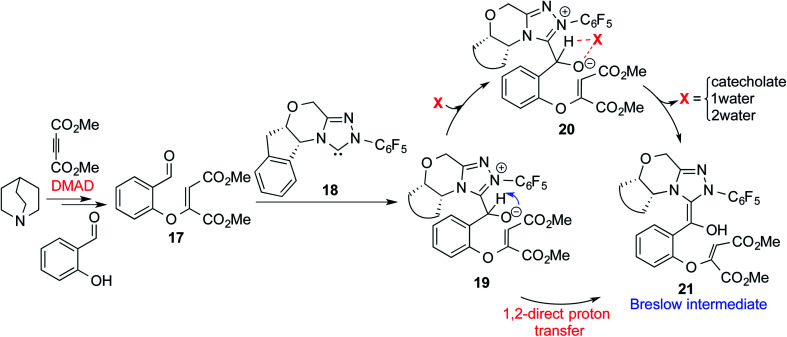
Key steps involved in the formation of the Breslow intermediate in a dual-catalytic Michael–Stetter cascade reaction.

In another recent study, Sunoj and co-workers reported the mechanism and origin of enantioselectivity in an asymmetric Michael–Stetter cascade reaction catalyzed by a chiral NHC and quinuclidine leading to the formation of benzofuran (R3, Table 1).^9*f*^ The energetically preferred pathway obtained at the SMD_(toluene)_/M06-2X/6-31+G** level of theory was suggested to begin with a nucleophilic addition of quinuclidine to DMAD as shown in Scheme 5. A few more ensuing steps (not of immediate relevance to the current discussion) furnish a DMAD–salicylaldehyde Michael adduct (**17**).

**Fig. 2:**
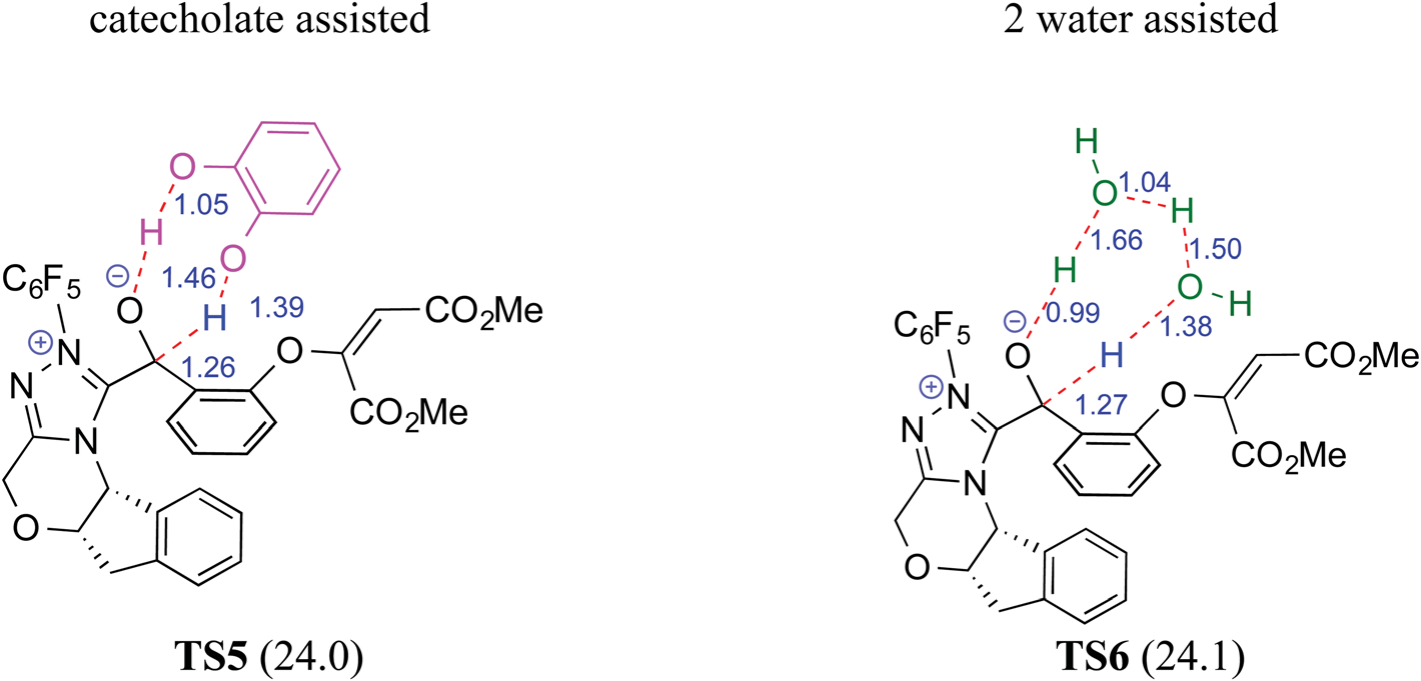
Key features of the transition state geometries for the assisted proton transfer involved in the formation of the Breslow intermediate. The activation barriers (Δ*G*^‡^ in kcal mol^−1^) at the SMD_(toluene)_/M06-2X/6-31+G**//M06-2X/6-31+G** level of theory are provided in parentheses. Distances are in angstroms.

The participation of an NHC was invoked in the following step wherein the NHC reacts with the aldehydic end of the salicylate intermediate **17** to form a zwitterionic species **19**. As with previous cases, a crucial proton transfer would convert **19** to the desired Breslow intermediate **21**. Here, a 1,2-direct proton transfer was predicted to encounter a barrier of 48.0 kcal mol^−1^ prompting the authors to consider two types of assisted pathway, one with the involvement of the additive catechol and the other with an explicitly included water molecule. It was noted that catechol assists the proton transfer through a stepwise process involving the catecholate as the key species (formed by the action of quinuclidine on catechol). Shown in Fig. 2 is a qualitative depiction of a concerted transition state (**TS5**), wherein the alkoxy oxygen abstracts the phenolic proton of the catecholate while the proton on the alkoxy carbon is simultaneously abstracted by the phenoxide end of the catecholate. The predicted barrier was 24.0 kcal mol^−1^ for the catecholate assisted proton transfer, half of that for the 1,2-direct proton transfer. They also evaluated the efficacy of water assisted proton transfer en route to the Breslow intermediate. The energy of relay proton transfer, assisted by two explicit water molecules, as shown in **TS6**, was incidentally the same as that of the catechol assisted pathway. The barrier for the assisted proton transfer, mediated by one water molecule, was found to be 32.1 kcal mol^−1^ (not shown in Fig. 2 for brevity and easier comprehension of the most likely alternatives). Therefore, the mechanism of formation of the Breslow intermediate in this example is more likely to involve an assisted proton transfer pathway, facilitated by the explicit participation of one catechol or two water molecules.

### (C) Base assisted pathway

**Fig. 3:**
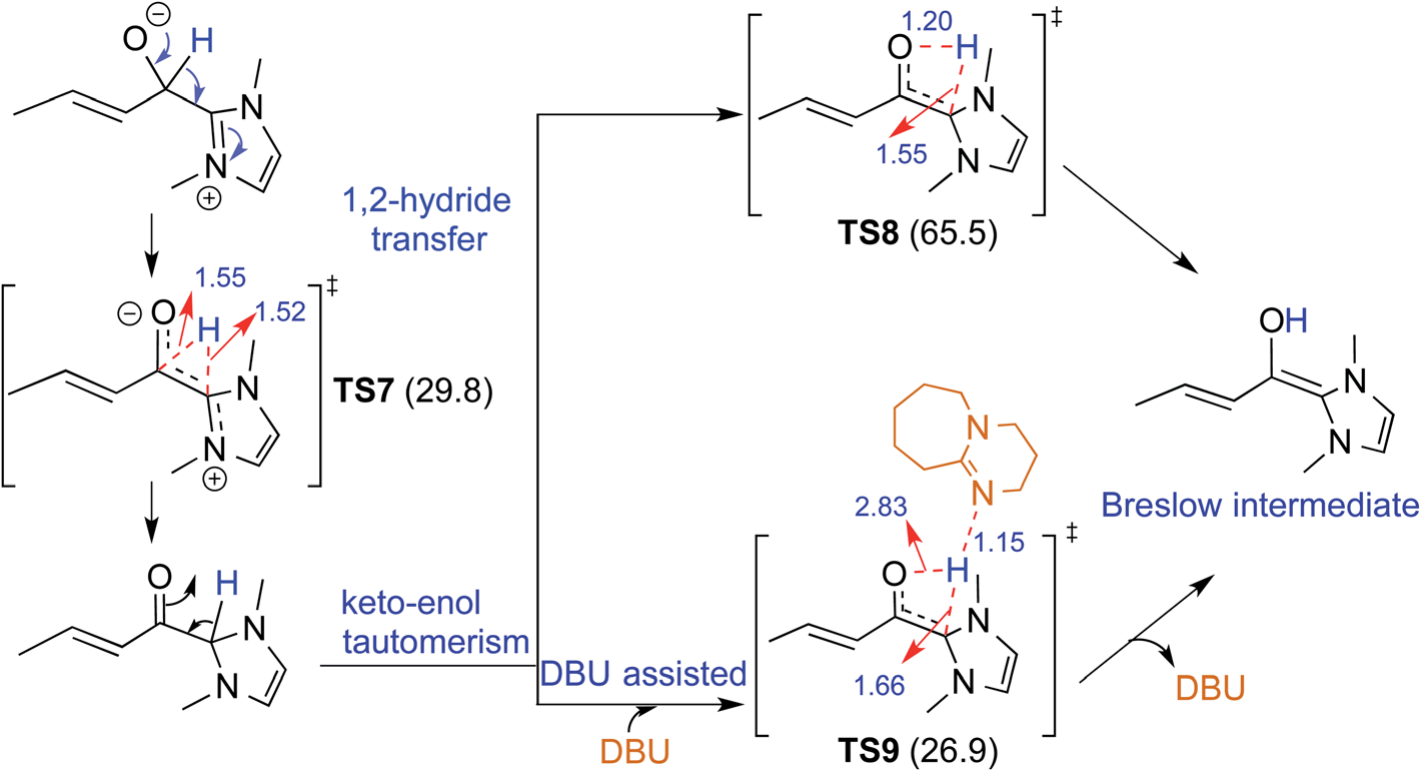
Key features of the transition states involved in the keto–enol pathway for the formation of the Breslow intermediate. The activation barriers (Δ*G*^‡^ in kcal mol^−1^) obtained at the PCM_(THF)_/M06-2X/6-311+G**//M06-2X/6-31+G** level of theory are shown in parentheses. Distances are in angstroms.

There have been reports that invoked the participation of bases such as DBU, KO^*t*^Bu, K_2_CO_3_, Na_2_CO_3_, Cs_2_CO_3_, and DABCO in the mechanism of formation of the Breslow intermediate.^10*d*–*i*,13^ A recent computational study from our laboratory has demonstrated the importance of a critical 1,2-hydride transfer under basic reaction conditions (R4, Table 1).^10*d*^ The barrier for the 1,2-hydride transfer *via***TS7**, as shown in Fig. 3, to form a keto-type intermediate was found to be about 30.0 kcal mol^−1^ at the PCM_(THF)_/M06-2X/6-311+G**//M06-2X/6-31+G** level of theory. A subsequent keto–enol tautomerization *via***TS8** was suggested toward the formation of the desired Breslow intermediate. A high barrier for this unassisted keto–enol tautomerization prompted the authors to consider a DBU assisted relay proton transfer *via***TS9**. Interestingly, the barrier was found to be about 27 kcal mol^−1^ for the DBU assisted pathway wherein DBU abstracts the proton from the NHC carbon and delivers it to the enolate oxygen. As would become more apparent in the ensuing sections of this article, both the keto and enol variants of the Breslow intermediate are inherently appealing and thus deserve additional attention, primarily owing to the ease of detection of the former (*vide infra*).

**Scheme 6:**
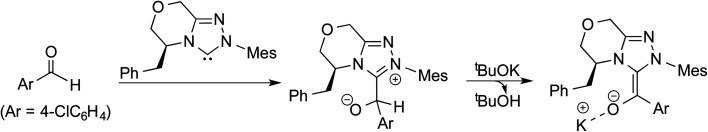
Formation of the potassium bound Breslow intermediate.

**Fig. 4:**
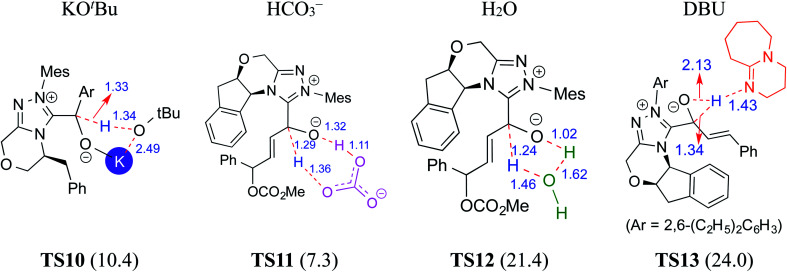
Key features of the transition states of various assisted proton transfers involved in the formation of the Breslow intermediate. The activation barriers (Δ*G*^‡^ in kcal mol^−1^) are shown in parentheses. Distances are in angstroms.

In another density functional theory study on a chiral NHC catalyzed intermolecular Stetter reaction (R5, Table 1),^10*e*^ the Sunoj group has identified that KO^*t*^Bu played a pivotal role in the formation of the Breslow intermediate. As with the previous examples, once the initial adduct between the aldehyde and NHC is generated, a proton transfer in the zwitterionic intermediate can take place through (a) a 1,2-direct process, or (b) a KO^*t*^Bu assisted pathway (Scheme 6). The authors noted an explicit participation of the base, where the potassium ion is bound to the alkoxide oxygen while the butoxide counterion is hydrogen bonded to the benzylic proton (Fig. 4), as kinetically beneficial. The barrier associated with this kind of assisted proton transfer transition state **TS10** was more than 40 kcal mol^−1^ lower than the unassisted direct proton transfer transition state at the SMD_(toluene)_/M06-2X/6-31G** level of theory. Additional analysis of the geometric features by using the intrinsic reaction coordinate computations on **TS10** indicated that the *t*-butoxide first abstracts the proton from the enolate carbon to generate ^*t*^BuOH and a potassium ion, thus leading to a potassium bound Breslow intermediate.

In 2015, Tang and co-workers reported the mechanism and enantioselectivity of an NHC catalyzed [4 + 2] annulation reaction between enals and azodicarboxylates in the presence of K_2_CO_3_ as the base (R6, Table 1).^10*f*,14^ The generation of the active catalyst from the pre-catalyst was suggested to take place by the action of the base. Similar to the earlier studies, three likely pathways for the 1,2-proton transfer, through unassisted and assisted modes (bicarbonate or H_2_O), were examined (Fig. 4). In the HCO_3_^−^ assisted pathway, one end of the HCO_3_^−^ abstracts the enolate C–H proton and transfers its proton to the alkoxide oxygen at the other end as depicted in **TS11**, in a concerted asynchronous manner. The Gibbs free energy barrier for **TS11** in the HCO_3_^−^ assisted proton transfer was 7.3 kcal mol^−1^ at the IEF-PCM_(THF)_/M06-2X/6-31G** level of theory, which is obviously far more favorable than the direct proton transfer process (42.4 kcal mol^−1^).^10*f*^ Similarly, the barrier for the water assisted proton transfer *via***TS12** was found to be 21.4 kcal mol^−1^, higher than that for the HCO_3_^−^ assisted pathway. These data suggest that the kinetics of Breslow intermediate formation is likely to be sensitive to the nature of base employed during the course of its generation.

In another computational study on a cooperative NHC–LiCl catalyzed reaction toward the synthesis of spirooxindole lactone (R7, Table 1),^10*g*^ Sunoj and co-workers found that a DBU-assisted 1,2 proton transfer in the zwitterionic intermediate (derived from cinnamaldehyde by the addition of an NHC) is critical to the formation of the Breslow intermediate. In this process, the basic nitrogen of DBU abstracts the C–H proton and subsequently transfers it to the alkoxide oxygen in a concerted manner (**TS13** in Fig. 4). As in the earlier examples presented in this review, the DBU assisted proton transfer (24.0 kcal mol^−1^) was energetically more preferred over the 1,2-direct proton transfer with a prohibitively large barrier (46.4 kcal mol^−1^) at the SMD_(THF)_/B3LYP-D3/6-31G** level of theory. Through all the above-mentioned examples, we hope to have conveyed the significance of the base assisted proton transfer in the formation of the Breslow intermediate.

### (D) Acid assisted pathway

**Fig. 5:**
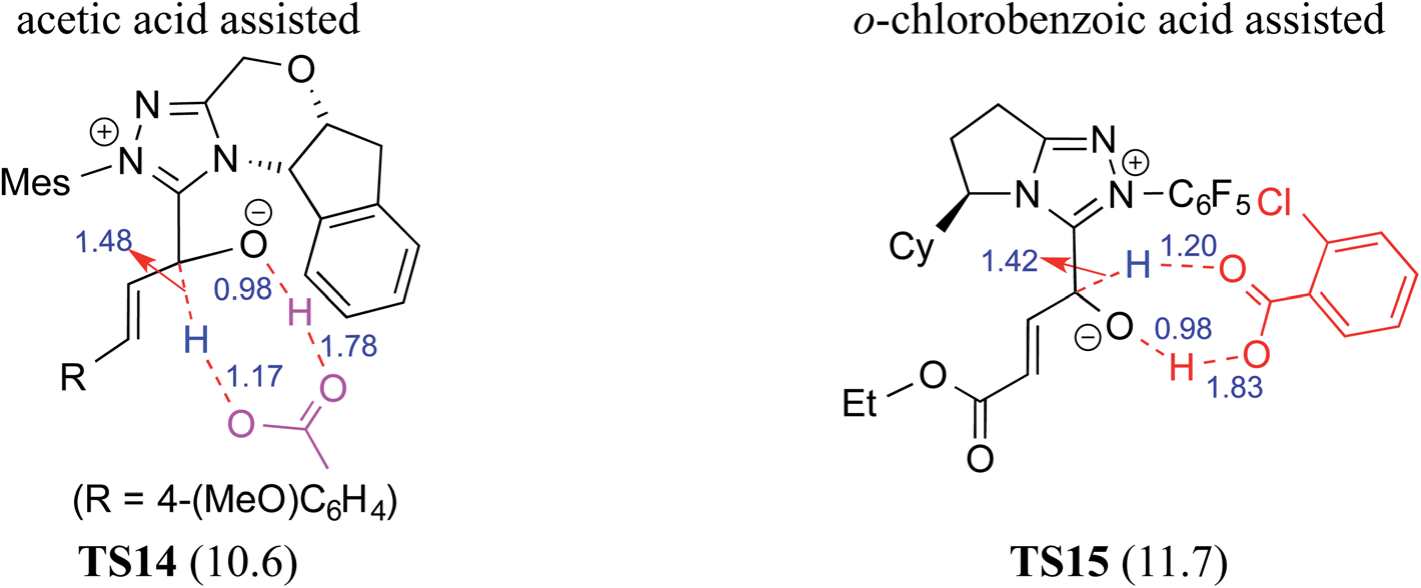
The key features of the transition state geometries of AcOH assisted and *o*-chlorobenzoic acid assisted seven-membered proton transfer involved in the formation of the Breslow intermediate. Activation barriers (Δ*E*^‡^ in kcal mol^−1^ for the AcOH assisted pathway and Δ*G*^‡^ for the chlorobenzoic acid assisted pathway) are shown in parentheses. Distances are in angstroms.

Acids such as acetic acid, benzoic acid, and other Brønsted acids have been employed as additives in various NHC catalyzed asymmetric reactions.^9*k*,15^ Acid additives have generally been considered as being capable of lowering the barrier for the formation of Breslow intermediates.^9*l*,10*j*,*k*,16^ Recently, Wei and co-workers have examined the mechanistic features and stereoinduction of a chiral NHC catalyzed [4 + 2] cycloaddition reaction at the IEF-PCM_(THF)_/B3LYP/6-31G** level of theory (R8, Table 1).^10*j*^ They noted that a seven-membered transition state for the acid assisted proton transfer (**TS14**, Fig. 5) had a lower barrier (10.6 kcal mol^−1^) than the corresponding five-membered alternative (16.7 kcal mol^−1^, not shown in the figure) as well as that in a direct proton transfer pathway (36.1 kcal mol^−1^).^17^ In the acid assisted proton transfer transition states, a stronger O–H⋯O hydrogen bonding interaction was noted in the seven-membered case than in the five-membered analogue.

**Fig. 6:**
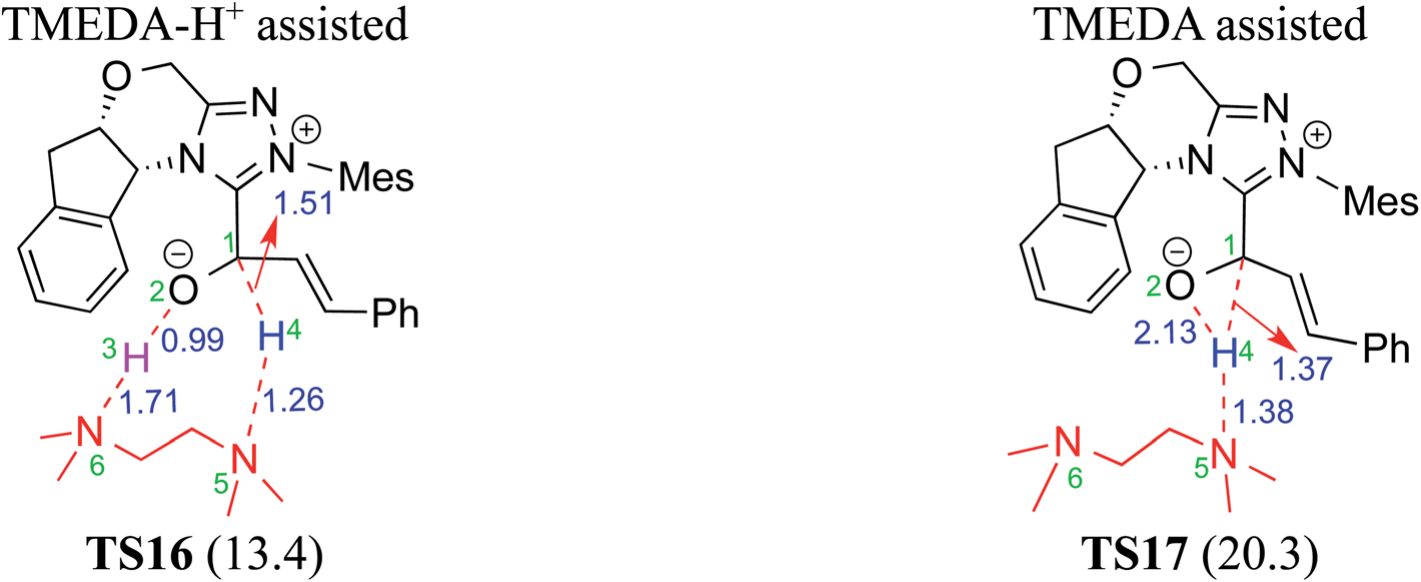
Key features of the transition state geometries of TMEDA-H^+^ and TMEDA assisted proton transfer in the formation of the Breslow intermediate. The free energy barriers (kcal mol^−1^) obtained at the IEF-PCM_(DCM)_/M06-2X/6-31G(d,p) level of theory are shown in parentheses. Distances are in angstroms.

Very recently, Sunoj and co-workers have examined the mechanism and origin of stereoinduction in cooperative asymmetric catalysis involving an NHC and Brønsted acid yielding γ-lactams by using SMD_(CH_3_CN)_/M06-2X/6-31G** computations (R9, Table 1).^9*l*^ In the initial step, sodium benzoate abstracts the proton from the triazolium salt to generate a free carbene and benzoic acid. The role of the *in situ* generated benzoic acid in the formation of the Breslow intermediate was disclosed. The Gibbs free energy barrier for the seven-membered benzoic acid assisted proton transfer transition state (**TS15**) was 11.7 kcal mol^−1^ (Fig. 5). The benzoic acid assisted proton transfer had a much lower barrier than the unassisted 1,2-direct proton transfer (38.7) as well as that involving an alternative five-membered transition state geometry (15.5 kcal mol^−1^, not shown). Benzoic acid assisted proton transfer was found to be a concerted process as confirmed through IRC calculations. In another interesting study, Tang and co-workers probed the mechanism of a cooperative catalytic protocol involving an NHC and a Brønsted acid (TMEDA-H^+^) in a [3 + 2] annulation between enals and α-ketoamides (R10, Table 1).^10*k*^ On the basis of the Gibbs free energy barriers obtained at the IEF-PCM_(DCM)_/M06-2X/6-31G(d,p) level of theory (Fig. 6), the Brønsted acid assisted proton transfer mechanism (**TS16**, 13.4 kcal mol^−1^) was suggested to be far more favorable than the TMEDA assisted (**TS17**, 20.3 kcal mol^−1^) as well as an unassisted proton transfer (44.3 kcal mol^−1^) route to the Breslow intermediate. It was noted that in **TS16** the proton transfer from TMEDA-H^+^ is almost complete (O2–H3 = 0.99 Å, N6–H3 is 1.71 Å) as compared to the removal of C1–H4 (1.51 Å) by the amino end of the protonated base. Importantly, the O2–H3⋯N6 hydrogen bonding interaction stabilizes both the intermediate and transition states (**TS16**). The overall process was highly exergonic and irreversible. Furthermore, the barrier for the abstraction of the H4 proton by N5 of TMEDA *via***TS16** was 13.4 kcal mol^−1^, which in turn leads to the Breslow intermediate and TMEDA-H^+^.

**Fig. 7:**
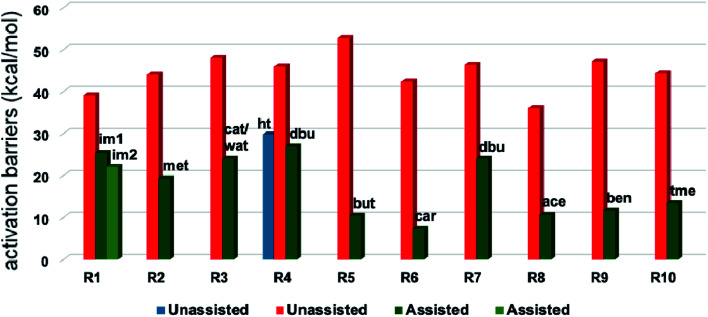
Graphical representation of Gibbs free energy barriers for the formation of the Breslow intermediate under different reaction conditions listed in Table 2. The red color in the bar diagram represents direct proton transfer and green represents acid/base/water/other species assisted proton transfer. Here, ‘ht’ represents 1,2-hydride transfer in reaction R4.

A summary of the activation barriers for the formation of Breslow intermediates, under different reaction conditions, is compiled in Table 2 and Fig. 7. It can readily be gleaned that the unassisted proton transfer generally encounters a prohibitively higher barrier than the alternative assisted pathway. Assisted pathways involving different acids (R8 and R9), bases (R4, R5, R6 and R7), water (R3 and R6), alcohols (R2 and R3), or other species (R1) were reported to facilitate easier proton transfer in the initially formed zwitterionic adduct between the NHC and substrate such as an aldehyde. Various two-step mechanisms, such as a bimolecular pathway, were also suggested for the proton transfer between the initially formed zwitterionic intermediate as a way to lower the barrier for the 1,2-direct proton transfer (in respect of R1).^10*a*^ The barrier for the first proton transfer between two zwitterionic intermediates was 25.4 kcal mol^−1^ while that for the second proton transfer was 22.1 kcal mol^−1^. A two-step process involving the initial formation of a zwitterionic intermediate and a subsequent 1,2-hydride transfer to a keto-type intermediate was also noted in reaction R4. The barrier for DBU-assisted proton transfer in the keto–enol tautomerization was found to be much lower (27.0 kcal mol^−1^) as compared to that for the corresponding unassisted alternative (65.5 kcal mol^−1^).^10*d*^ Although this two-step mechanism of formation of Breslow intermediates was proposed in certain systems, it should be noted that the single step pathways are energetically more feasible (*e.g.*, R2, R3, R5, R6, R7, R8, and R9). Proton transfer assisted by K_2_CO_3_ was reported as energetically more favorable than other bases, acids, water, or alcohol. These findings collectively indicate that the choice of additive is likely to have a direct bearing on the efficiency of formation of the Breslow intermediate and hence on its subsequent catalytic abilities as well. Apart from the environmental influences exerted by various additives on the energetics of formation of Breslow intermediates, it would also be important to consider intrinsic factors such as the nature of the NHC and the substituents therein. In the following section, we wish to present one such interesting example for specific attention and more in the latter part of this thesis.

**Table 2:** The computed barriers for assisted proton transfers involved in the formation of Breslow intermediates with different NHCs for a selected set of reactions

Reaction^a^	Acid/base/water/other species	Notation	Δ*G*^‡^ (kcal mol^−1^)
R1	Zwitterionic intermediate (step 1)	im1	25.4
	Zwitterionic intermediate (step 2)	im2	22.1
R2	MeOH	met	19.3
R3	H_2_O	wat	24.0
	Catechol	cat	24.0
R4	DBU	dbu	26.9
R5	KO^*t*^Bu	but	10.4
R6	K_2_CO_3_	car	7.3
R7	DBU	dbu	24.0
R8	AcOH	ace	10.6^b^
R9	*o*-Chlorobenzoic acid	ben	11.7
R10	TMEDA	tme	13.4

a See Table 1 for details about reactions R1, R2, …, R10.

b Δ*E*^‡^.

## Effect of *N*-aryl substituent on Breslow intermediate formation

**Scheme 7:**
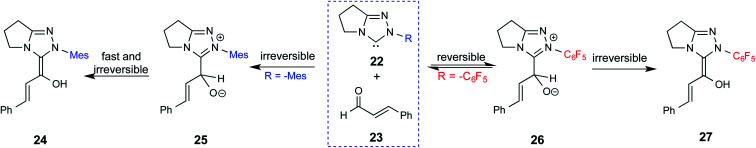
Effect of the *N*-aryl substituent on the Breslow intermediate formation.

Since the early years of their development, it has widely been recognized that the substituents on the imidazolidine nitrogen play a vital role in the stability as well as electronic properties of NHCs. Hence, it is conspicuously important to the present discussion to establish the role of *N*-substituents in the formation of Breslow intermediates. The geometric proximity and the ability of such substituents in altering the electron donating ability of the nitrogen to the carbenic carbon are of significance to the energetics of formation of the Breslow intermediate. One such study that sheds light on the effect of *N*-aryl substituent in NHC catalyzed reactions was from the Bode group. Noteworthy conclusions gathered from their control experiments and kinetic measurements conveyed that the Breslow intermediate formation is irreversible with *N*-mesityl carbene while with the N–C_6_F_5_ analogue it is reversible (Scheme 7).^18^ Furthermore, the nature of the *N*-aryl substituent was suggested to impact the overall kinetic features of the catalytic transformation. For instance, the Breslow intermediate formation was noted as the rate determining step in the case of *N*-mesityl NHC while the subsequent nucleophilic addition of the Breslow intermediate to the substrate enone was found to be the rate determining step with N–C_6_F_5_ substituted NHC.

For many years, the participation of the Breslow intermediate in NHC catalyzed reactions remained a mechanistic hypothesis with a long status of being a putative species. With the ever-increasing popularity of NHC catalyzed reactions, interest toward the isolation and characterization of Breslow intermediates became a thriving area of activity. A repertoire of experimental tools, such as X-ray crystallography, IR, NMR, and UV-Vis spectroscopy, have been employed as applicable in the solid state and in solution as well as in the gas phase to gather information on important features of Breslow intermediates. In the following section we describe some of the quintessential efforts made in this front.

## Characterization of the Breslow intermediate

**Fig. 8:**
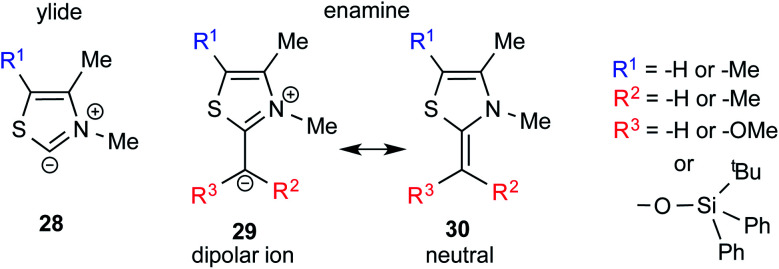
Key intermediates involved in thiamine diphosphate dependent enzymatic pathways, which can be regarded as a structural analogue of Breslow intermediates.

One of the earliest reports on the characterization of motifs similar to the Breslow intermediates comes from a seemingly different domain of enzymatic catalysis. In the late 80s, Jordan and co-workers synthesized alkyl thiazolium species (**29** and **30**) through the reaction between alkyl halides and thiazolium salts, which are a close analogue of the Breslow intermediate (Fig. 8). They also reported the structural analogues of enamine intermediates involved in enzymatic pathways and their characterization using ^1^H NMR spectroscopy.^19^

**Scheme 8:**
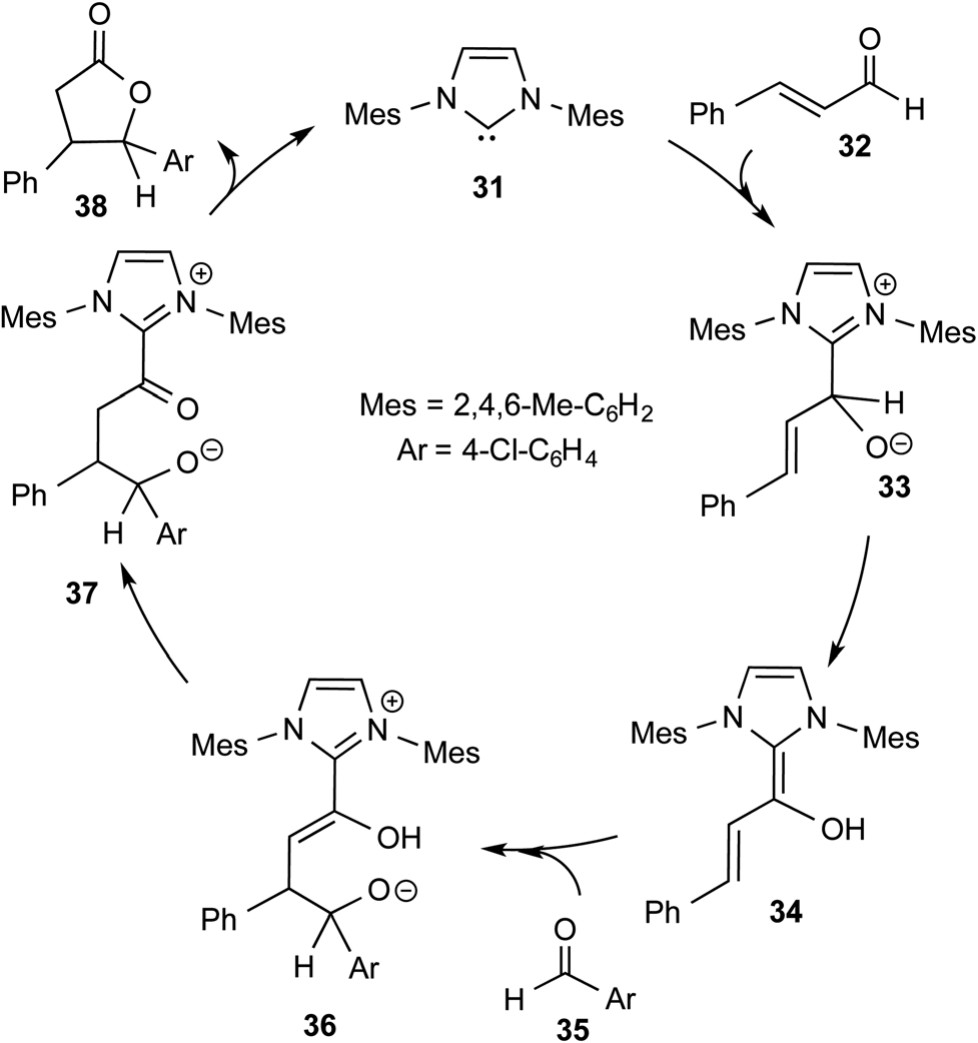
Proposed mechanism for the N-heterocyclic carbene catalyzed reaction between cinnamaldehyde and 4-chlorobenzaldehyde to form a γ-lactone.

More direct efforts toward the realm of characterization of Breslow intermediates began to appear in the later years by way of engaging carefully chosen substrates such as α,β-unsaturated aldehydes. In 2004, Glorius and Bode independently developed the fascinating concept of homoenolate equivalent, which was followed up by other researchers as well.^20^ This idea is illustrated by using a representative example of NHC catalyzed reaction between cinnamaldehyde and 4-chlorobenzaldehyde, as shown in Scheme 8.^21^ The proposed catalytic cycle involves the initial addition of NHC **31** to α,β-unsaturated aldehyde **32** to form a zwitterionic intermediate **33**, which on subsequent proton transfer yields a homoenolate equivalent in the form of the diamino dienol intermediate **34**. Species **34** exhibits interesting conjugate umpolung reactivity in its reaction with an aldehyde such as **35** to form a γ-lactone, **38**, through an intramolecular cyclization.

**Scheme 9:**
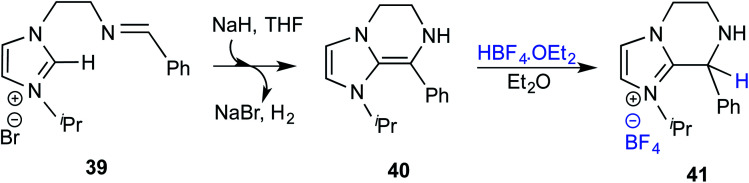
Intramolecular cyclization in the presence of a base to form a Breslow type intermediate, **40**.

The mechanistic proposal was supported by electrospray mass spectrometric measurements on samples drawn from the reaction mixture. The observed values of various *m*/*z* peaks revealed the identities of important intermediates. For instance, low intensity peaks pertaining to the protonated form of **33** and the diamino dienol intermediate **34** could be noted. These experiments offer compelling evidence for the participation of the Breslow intermediate under the given reaction conditions. The presence of a high intensity peak at *m*/*z* 305 emanating from the protonated catalyst **31** was also a valuable insight into the overall mechanism of NHC catalysis. In another recent study, Douthwaite and co-workers demonstrated the formation of a Breslow type intermediate, **40**, derived from unactivated imines through an intramolecular cyclization in the presence of a base (Scheme 9).^22^ Similar to a Breslow intermediate, the nucleophilic reactivity of an amino analogue such as **40** could be demonstrated through a simple protonation reaction as shown below.

**Scheme 10:**
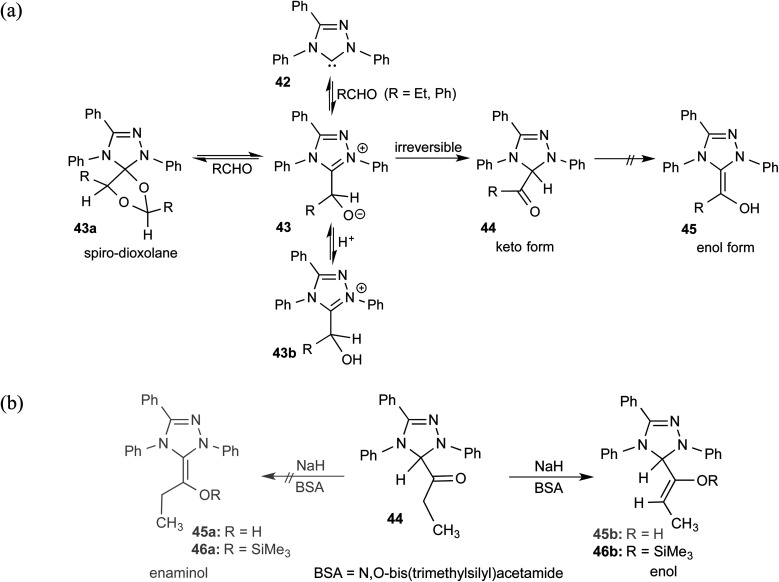
(a) Generation of the keto-tautomer and spiro-dioxolane from a triazolylidene carbene and an aliphatic aldehyde. (b) The formation of the keto-tautomer of the Breslow intermediate.

As we begin discussing the isolation and characterization of Breslow intermediates, it is prudent to acknowledge the pioneering contributions from the Berkessel group, by taking one of their examples as a starting point. As the earliest step in this front, back in 2010, they could perform NMR characterization of the keto variant of a Breslow intermediate^23^ derived from substituted triazolylidene carbenes **42** and an aliphatic aldehyde (Scheme 10(a)). It was noticed that a 1 : 1 molar ratio of the carbene and aldehyde exclusively results in the keto tautomer of the Breslow intermediate (**44**), while excess aldehyde led to a spiro-dioxolane (**43a**). The latter could potentially be a resting state in the catalytic cycle, from where dissociation to the monomeric form should take place for its active participation in catalysis. In the presence of an acid, the reversible formation of an isolable intermediate, **43b**, was noticed.

The B3LYP/6-31G(d,p) computations revealed that the enol intermediate **45** (Scheme 10(a)) is 14 kcal mol^−1^ higher in energy than the corresponding keto form **44** (when R = Ph). While the keto form was found to be catalytically inactive, attempts at tautomerization to the active enol form by using various acids, bases, and silylating agents also remained unsuccessful (Scheme 10(b)). The tautomerization of **44** to an enaminol (**45a**) or to an enol (**45b**) was in vain too, even in the presence of a catalytic amount of acid (TFA, *p*-Ts-OH). Interestingly, the use of a catalytic amount of NaH base in the presence of a silylating agent, BSA (*N*,*O*-bis(trimethylsilyl)acetamide), did provide access to a silylated enol ether, **46b**, while the formation of an active enaminol, **46a**, could not be established. It is likely that **46a** was either not formed or was not amenable to detection under the conditions employed.

**Scheme 11:**
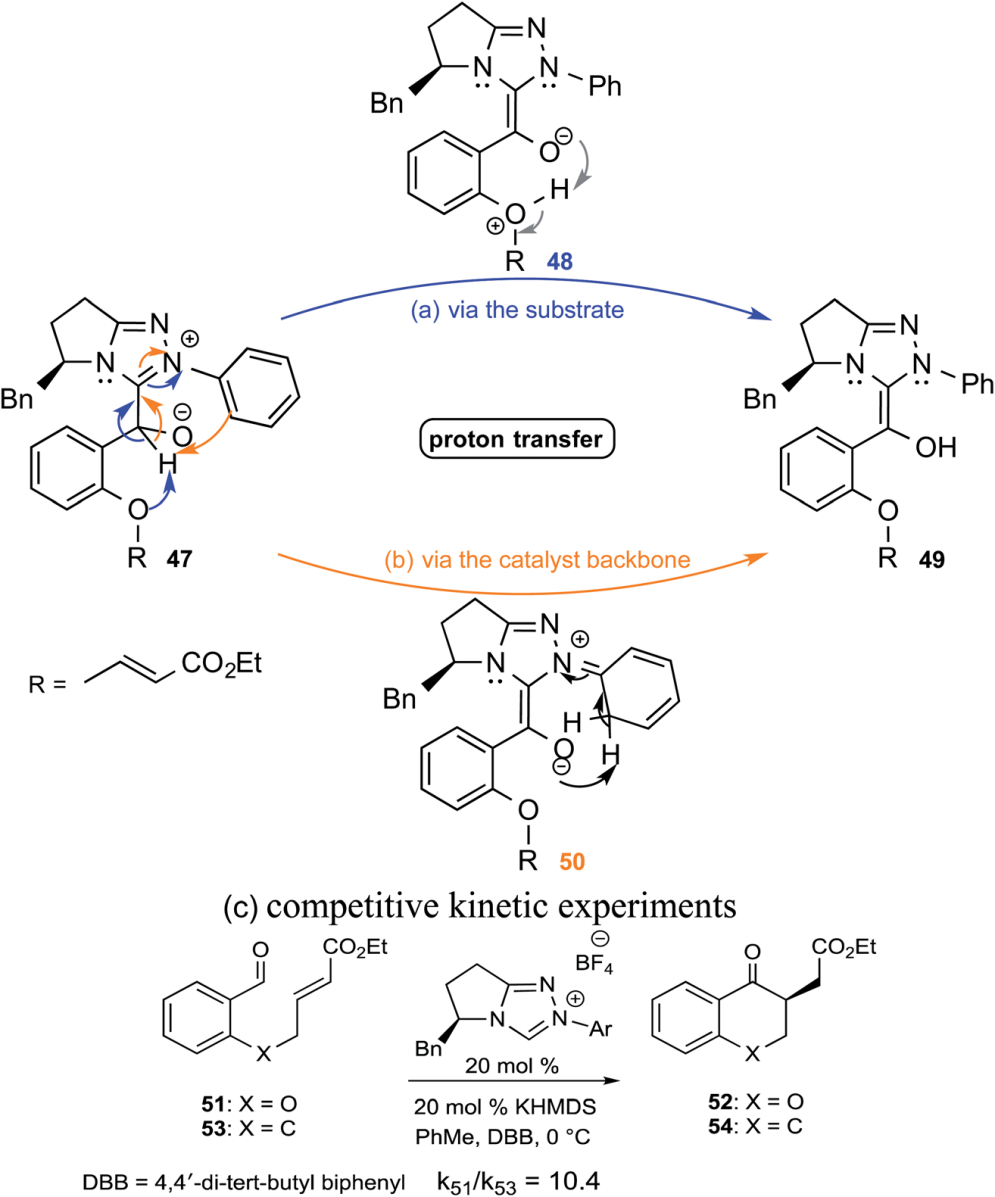
Likely proton transfer pathways as well as competitive experiments between aldehydes **51** and **53**.

In an interesting study on the triazolinylidene carbene-catalyzed asymmetric intramolecular Stetter reaction (Scheme 11), Rovis and co-workers noted that the proton transfer from the aldehydic carbon to the geminal oxygen is the first irreversible step in the mechanism of formation of the Breslow intermediate.^24^ Two key pathways for the proton transfer in the initially formed zwitterionic intermediate **47** were envisaged, (a) facilitated by the –OR group of the substrate (*via* intermediate **48**) and (b) by engaging the *N*-aryl group of the catalyst (involving **50**). Carefully designed kinetic experiments using substrates **51** and **53**, as shown in Scheme 11(c), could shed more light on the mechanism of 1,2-proton transfer. The faster reaction rate with **51** bearing an ether linkage was considered as evidence for an intramolecular substrate-assisted proton transfer (Scheme 11(a)). To test whether the *N*-aryl group of the catalyst was involved in the proton transfer, kinetic isotope effect studies were conducted by using deuterated aldehyde **47**. The absence of deuterium incorporation at the *ortho* position of the catalyst *N*-aryl group (in intermediate **50**) confirmed that the proton was not transferred *via* the catalyst as shown in Scheme 11(b). Interestingly, the formation of a cyclic product was also observed in the case of substrate **53** lacking an ether linkage (Scheme 11(c)), which is an indication that with such substrate an alternative proton transfer mechanism may operate.^10*a*^

**Scheme 12:**
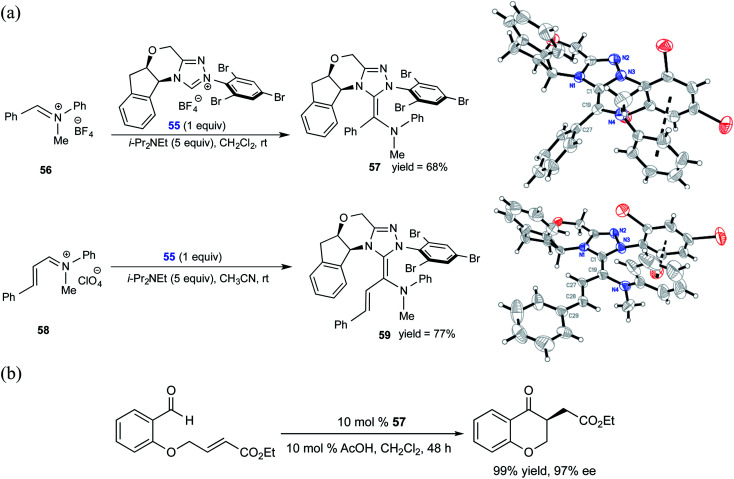
(a) Isolation and characterization of aza-analogues of Breslow intermediate **57** and a homoenolate analogue of Breslow intermediate **59**. (b) Stetter reaction catalyzed by an aza-Breslow intermediate. Reprinted with permission from ref. 25. Copyright 2012 American Chemical Society.

Similar to the interest in native Breslow intermediates, there have been efforts toward the synthesis of other analogues such as aza-Breslow intermediates. A recent report by the Rovis group is particularly noteworthy where they disclosed the synthesis, detection, and characterization of aza-Breslow intermediates using a combination of UV-Vis, NMR, and X-ray crystallography (Scheme 12) tools.^25^ This kind of evidence provides a firmer ground for the involvement of Breslow intermediates in NHC catalysis. Besides the detection and characterization of aza-Breslow intermediates (**57** and **59**), their own catalytic activity was demonstrated in an asymmetric Stetter reaction. Intermediates **57** and **59** are respectively an acyl anion and a homoenolate equivalent, which are typically seen in native NHC catalysis involving Breslow intermediates.

**Scheme 13:**
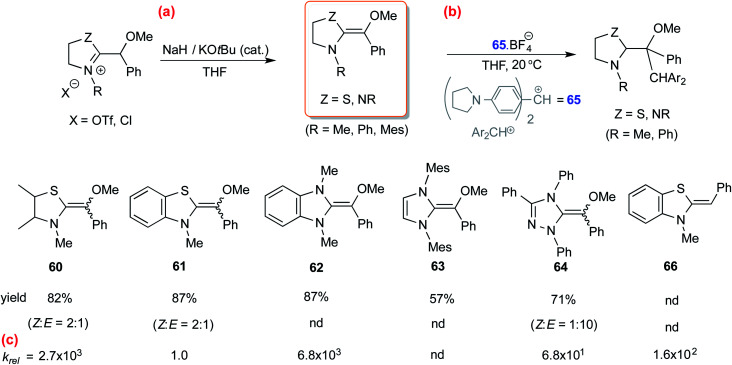
(a) Generation of *O*-methylated Breslow intermediates from the corresponding azolium salts, (b) their reactivities toward an electrophile, and (c) the relative rates.

In another study, Mayr and co-workers isolated an *O*-methylated Breslow intermediate by the deprotonation of the corresponding azolium salt in the presence of NaH and catalytic amounts of KO^*t*^Bu as shown in Scheme 13(a).^26^ The thiazole derived intermediates **60** and **61** were formed as a mixture of *Z* and *E* isomers (*Z*/*E* = 2 : 1) while triazole analogue **64** resulted in *Z*/*E* of 1 : 10 (Scheme 13(b)). An interesting structure–activity relationship was established by kinetic experiments using the stabilized benzhydrylium ion **65** as the electrophile (Schemes 13(b) and (c)) and a range of *O*-methylated Breslow intermediates as the nucleophilic partners. It was found that the thiazole *O*-methylated Breslow intermediate (**61**) was less nucleophilic than the corresponding deoxy-Breslow intermediate (**66**). The relative rates between **62** and **61** indicated that the *O*-methylated Breslow intermediate derived from thiazole was 2 to 3 orders of magnitude less reactive than the structurally analogous imidazole family.

**Scheme 14:**
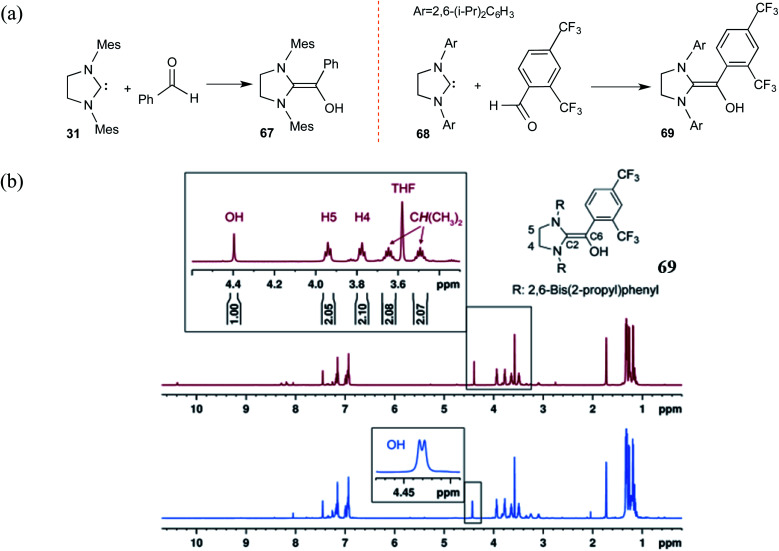
(a) Generation of Breslow intermediates by using saturated carbenes. (b) The ^1^H NMR spectrum of the diamino enol **69** showing the magnified region of the peaks pertaining to the carbene and the Breslow intermediate protons as marked in the structure. The blue color magnified peaks show the doublet of the diamino enol proton due to the adjacent ^13^C-labeled carbon. Reprinted with permission from ref. 27. Copyright 2012 John Wiley and Sons.

Along similar lines, Berkessel and co-workers reported the synthesis of diamino enols using saturated carbenes. The reaction of dihydro-imidazolidine-2,4,6-trimethylphenyl (SIMes) **31** or dihydro-imidazolidine-2,6-bis(2-propyl)phenyl (SIPr) **68** with benzaldehyde or 2,4-bis(trifluoromethyl)benzaldehyde, respectively, yielded Breslow intermediates **67** and **69** (Scheme 14(a)). A comparison of these adducts of triazolylidene (as shown earlier in Scheme 10) and that formed by dihydro-imidazolylidene, with benzaldehyde as the electrophile, would be of interest here. The former NHC resulted only in the keto form of the Breslow intermediate (**44**), while the latter offered access to the desired enaminol variants **67** and **69**. These Breslow intermediates were further examined using *in situ* NMR spectroscopy.^27^ A characteristic peak at *δ* = 4.40 ppm in the ^1^H NMR spectrum of **69** (Scheme 14(b)) was confirmed as arising from the diamino enol through rapid H/D exchange upon introducing [D_4_]MeOH. The use of ^13^C-labeled 2,4-bis(trifluoromethyl)benzaldehyde (^13^CHO) resulted in a coupling between ^13^C (labeled as C6) and the OH group (^2^*J*_C–OH_ = 3.23 Hz). The other characteristic ^13^C peaks observed were at *δ* = 145.8 ppm (^1^*J*_C6–C2_ = 106.4 Hz, C2) and *δ* = 142.5 ppm (^1^*J*_C6–C_ar__ = 69.8 Hz, C_ar_). These spectral features confirmed the formation of diamino enol between the saturated carbene SIPr and 2,4-bis(trifluoromethyl)benzaldehyde. These pieces of unequivocal evidence are noteworthy inroads toward a long-standing goal of establishing the true nature of Breslow intermediates.

**Scheme 15:**
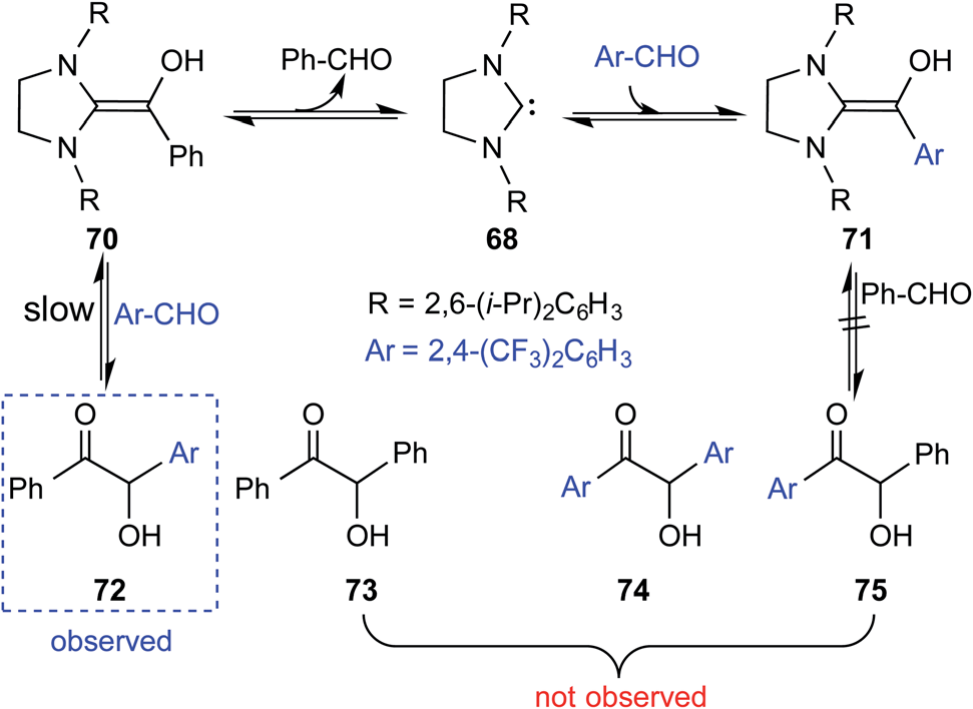
Crossover experiment for deducing the reversibility in the formation of the Breslow intermediate.

Interesting crossover experiments were performed to gather additional details regarding the equilibrium of Breslow intermediate formation (Scheme 15). It was noted that enaminol **70** exists in equilibrium with the free carbene **68** and benzaldehyde. The use of a more electrophilic 2,4-bis(trifluoromethyl) benzaldehyde exhibited rapid equilibrium between **70**, **68**, and **71**. The formation of a cross benzoin product (**72**) as the major product was considered as a tangible indication of a fully reversible equilibrium operating between Breslow intermediates **70** and **71**. Experiments of such kind that were able to identify an equilibrium between a free NHC and the corresponding Breslow intermediate do convey a gratifying message that our quest for studying enaminol intermediates has come a long way.

**Scheme 16:**
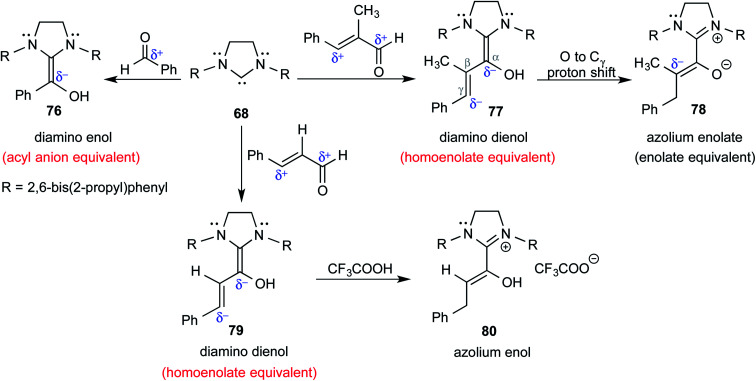
Formation of different likely reactive intermediates in the reaction between NHC and a simple aldehyde or an α,β-unsaturated aldehyde.

In keeping with their continued interest in Breslow intermediates, Berkessel and co-workers characterized a set of key intermediates involved in the NHC catalyzed umpolung reactions of various aldehydes by using X-ray crystallography (Scheme 16).^28^ Intermediates such as diamino enol **76** (acyl anion equivalent), diamino dienols **77** and **79** (homoenolate equivalent), azolium enolate **78** (enolate equivalent), and azolium enol **80** could be identified. Each of these intermediates hold a prominent place in NHC catalysis as they are known to react with a range of different electrophiles to furnish a good number of valuable products.

**Scheme 17:**
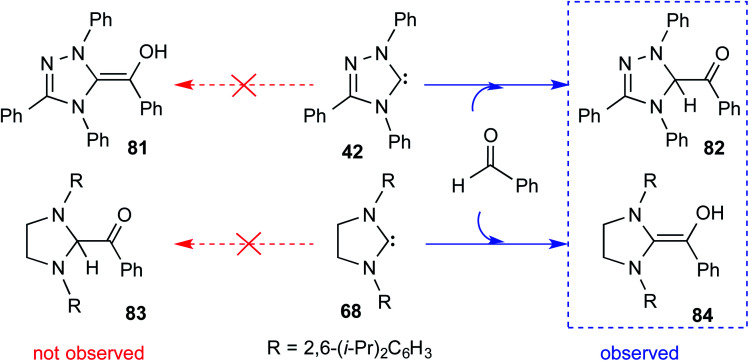
Formation of the keto and enol forms of the Breslow intermediate with triazolylidene and imidazolylidene carbenes with benzaldehyde, respectively.

Although the isolation and characterization of Breslow intermediates, derived from saturated dihydro-imidazolylidene and aldehydes/enals, were successful, certain puzzling observations pertinent to the formation of Breslow intermediates remained open. For instance, an exclusive formation of the keto form of the Breslow intermediate **82** was noticed in the case of triphenyltriazolylidene carbenes (**42**), while the saturated imidazolylidene carbenes (**68**) yielded the enol tautomer (**84**) in their stoichiometric reaction with benzaldehyde (Scheme 17).^29^

**Scheme 18:**
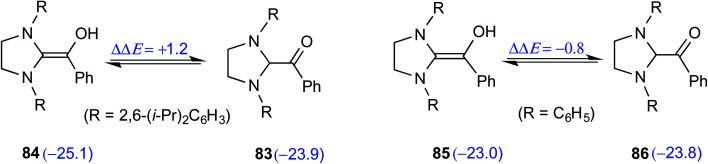
The relative energies of the enol and keto (shown in parentheses, in kcal mol^−1^) variants of the Breslow intermediate with different aryl groups on the nitrogen atoms of imidazolylidene carbene.

The electronic energy computed at the M06-2X-D3/def2-TZVPP/IEFPCM_(THF)_//M06-L-D3/6-31+G(d,p) level of theory for the enol was found to be 2 kcal mol^−1^ lower when 2,6-bis(iso-propyl)phenyl was the *N*-R substituent as compared to that of a simple phenyl substituted NHC (Scheme 18). The electronic energy for the conversion of the enol to the ketone form for both **84** and **85** was predicted to be similar even as the *N*-aryl substituents were different. After the success with diamino enols derived from the saturated imidazolidin-2-ylidenes, efforts were expended toward studying aromatic NHCs such as benzimidazolin-2-ylidenes and thiazolin-2-ylidenes. Interestingly in 2019, Berkessel and co-workers could crystallize Breslow intermediates derived from aromatic thiazolin-2-ylidenes and subject them to XRD analysis. Furthermore, solution-phase NMR spectroscopic characterization of another Breslow intermediate obtained from aromatic thiazolin-2-ylidenes and an aliphatic aldehyde (trifluoroacetaldehyde) could also be accomplished.^30^

**Scheme 19:**
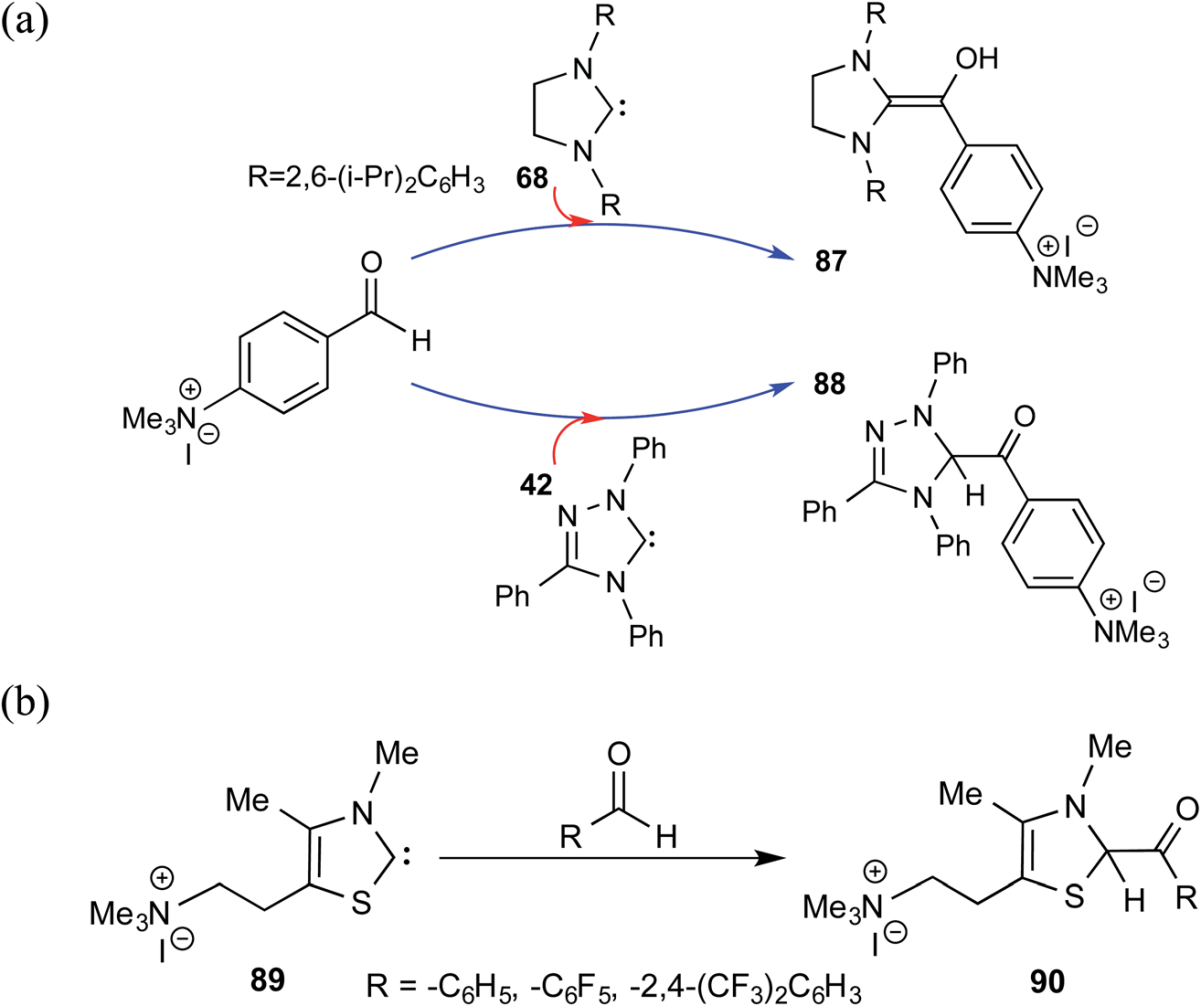
The Breslow intermediate derived from imidazolidin-2-ylidene with ammonium charge-tagged benzaldehyde and the corresponding keto analogue obtained using thiazolin-2-ylidenes.

After their successful series of reports on the characterization of various Breslow intermediates, both in the solution and in the solid state, Berkessel and co-workers have most recently disclosed a gas phase study of an ammonium charge-tagged Breslow intermediate by using ESI-MS IR ion spectroscopy in conjunction with quantum-chemical calculations.^31^ The ammonium charge-tag was introduced either on the NHC or aldehyde moiety, as shown in Scheme 19. The Breslow intermediate derived from the imidazolidin-2-ylidene **68** existed as a diamino enol, **87**. The presence of IR stretching frequencies at 1538 cm^−1^ (experimental) and 1524 cm^−1^ (computed) was attributed to the enol C=C(OH) functionality of diamino enol **87**. The detection of diamino enol was in concert with the computed energetic preference of 12 kcal mol^−1^ toward **87** as compared to the corresponding keto form at the M06-2X-D3/def2-TZVPPD//M06-L-D3/6-31+G(d,p) level of theory. On the other hand, attempts to obtain the Breslow intermediate using 1,2,4-triazolin-5-ylidenes **42** and thiazolin-2-ylidenes **89** resulted in keto structures, shown respectively as **88** and **90**. Both the experimental and calculated IR spectra showed the presence of a carbonyl C=O stretching band at 1720 cm^−1^ and at 1670 cm^−1^ for **88** and **90**, respectively.

## Summary and outlook

Breslow intermediates occupy a unique position in the contemporary literature owing to the remarkable popularity of N-heterocyclic carbene (NHC) catalysis. In due cognizance of the central role of Breslow intermediates in a myriad of NHC organocatalytic reactions and the lack of a single consolidated resource on their formation, isolation, and characterization, we have compiled the available knowledge in this review and have provided a critical assessment of their developmental strides. As the title implies, this treatise is philosophically organized from ‘then’ to ‘now’, and thus provides chronological details of the evolution of Breslow intermediates.

The age-old concept of umpolung, invoked for understanding the homo-dimerization of aldehydes in the presence of cyanide, bears noteworthy semblance to certain catalytic protocols employed in recent times. The suggested role of NHCs in promoting a similar umpolung reactivity became a conspicuous landmark when Ukai and co-workers identified that thiazolium salts could catalyze the benzoin reaction. A few decades later, Breslow proposed the involvement of a nucleophilic amino enol intermediate in the mechanism of a thiazolium catalyzed benzoin reaction. While this putative intermediate remained rather dormant for long, the renaissance in organocatalysis in the last two decades appears to have provided the much-needed impetus. Obviously, recent years witnessed an unprecedented series of applications of such amino enols in NHC catalyzed organocatalytic reactions. In honor of his pioneering work, amino enols have been termed as “Breslow intermediates”.

In the years that followed, efforts toward the generation of structural analogues of Breslow intermediates became an attractive endeavor. The characterization of Breslow intermediates derived from saturated imidazolylidene, unsaturated benzimidazolin-2-ylidenes, and thiazolin-2-ylidenes was realized. The electronic features of the *N*-substituents on the carbene backbone were identified to influence the formation as well as the subsequent reactivity of nucleophilic Breslow intermediates under different reaction conditions. Experimental endeavors probing the formation of Breslow intermediates were ably complemented by computational studies that helped in gaining key molecular insights. Importantly, computations revealed that after the initial addition of the NHC to a suitable enal/enone or similar electrophilic partner, the ensuing proton/hydride transfer encountered high barriers. A direct proton transfer in the initial zwitterionic intermediate through a strained three-membered transition state, or a 1,2-hydride transfer first to a keto intermediate that tautomerizes *via* a strained four-membered transition state to the corresponding Breslow intermediate, had a prohibitively high kinetic barrier. Computational studies demonstrated that the vital proton transfer could be assisted by an explicit participation of additives/solvent. Assisted proton transfer promoted by various additives (such as K_2_CO_3_) exhibited lower barriers than the corresponding unassisted pathways in the formation of Breslow intermediates, with a general preference for the assisted 1,2-proton transfer than the alternative keto–enol mechanism. Hence, the single step mechanism of formation of Breslow intermediates, with barriers in the range of 7 to 24 kcal mol^−1^ (depending on the molecular system), was recommended as more favorable over the two-step mechanism (with the overall barriers between 25 and 27 kcal mol^−1^).

Besides the work on the energetic origin of formation of Breslow intermediates, inexorable efforts led to successful elucidation of their structure by using X-ray crystallography in the solid state, NMR studies in the solution phase, and more recently ESI-MS IR ion spectroscopy in the gas phase. While our ability to perform *in situ* detection of Breslow intermediates under a plethora of diverse reaction conditions has significantly improved, certain subtle issues related to the energetics of formation that leads to the keto form with imidazolylidene carbenes and enaminol variants with dihydro imidazolylidene seem to demand more careful scrutiny. In summary, we have enunciated the state-of-the-art understanding on the formation and nature of Breslow intermediates in NHC organocatalysis, which is expected to serve as a single source of valuable knowledge on this important topic.

## Author contributions

All authors conceptualised the review, M. P. and Y. R. wrote the first draft supervised by R. B. S., who edited the manuscript.

## Conflicts of interest

There are no conflicts of interest to declare.
